# Supplementary material for: Dietary Polysaccharide-Rich Extract from Noni (Morinda citrifolia L.) Fruit Modified Ruminal Fermentation, Ruminal Bacterial Community and Nutrient Digestion in Cashmere Goats
Source: Animals (Basel). 2023 Jan 6;13(2):221. doi: 10.3390/ani13020221 (PMC9854603; doi:10.3390/ani13020221)
Supplement: Supplementary file 1 [file animals-13-00221-s001.zip › animals-2014797-supplementary.pdf]

Supplementary Table S1. Detailed list of compounds in NFP

Compounds	Content（%）
Saccharide	45.47
Glucopyranoside	9.88
Glucoside	15.6
Fructofuranoside	0.188
Fructoside	4.00
Rhamnoside	0.173
Xyloside	0.013
Galactoside	1.25
Mannose and its derivatives	0.086
Glucuronic acid and its derivatives	7.14
Galacturonic acid and its derivatives	0.126
Lactose and its derivatives	0.457
Sucrose and its derivatives	0.735
Phenolic glucoside	4.65
Other glucoside	0.388
Oligosaccharide	0.243
Amylose	0.098
Others	0.439
Organic acids and their derivatives	21.37
Monocarboxylic acid	2.39
Dicarboxylic acid	0.079
Aliphatic organic acids	15.9
Aromatic organic acid	0.710
Furanic acid	0.512
Terpenoid acid	0.048
Others	1.77
Lipids	6.09
Fatty acids and their derivatives	1.42
Phospholipids	0.506
Glycolipid	0.108
Lactone	1.65
Aromatic ester	1.49
Fatty acid ester	0.915
Alkaloids and their derivatives	6.14
Tropane alkaloids	1.66
Furan alkaloids	3.01
Pyrrolidine alkaloids	0.141
Organic amines alkaloids	0.995
Others	0.339
Amino acids and their derivatives	7.06
Amino acid	0.440
Oligopeptides	0.208
Aliphatic group derivatives	2.51
Aromatic group derivatives	0.138
Sulfur-containing group derivatives	0.059
Basic group derivatives	1.95

Acid group derivatives	0.003
Amide group derivatives	1.33
Others	0.413
Coumarins	1.36
Simple coumarins	0.345
Pyranocoumarins	0.034
Isocoumarin	0.962
Others	0.016
Terpenes	1.18
Monoterpenes	0.183
Diterpenes	0.013
Triterpenes	0.312
Sesquiterpenoids	0.005
Saponins	0.255
Terpene ketones	0.155
Others	0.260
Cholines	1.22
Cholines	0.987
Phosphorylcholine and its derivatives	0.092
Phosphatidylcholine and its derivatives	0.137
Nucleotides and their derivatives	1.21
Adenosine and its derivatives	0.551
Cytarylate and its derivatives	0.254
Guanylate and its derivatives	0.220
Uridine acid and its derivatives	0.036
Others	0.152
Alcohols	1.13
Monohydric alcohols	0.114
Dihydric alcohols	0.042
Trihydric alcohols	0.051
Steroids	0.399
Fatty alcohols	0.328
Alkamines	0.113
Alkenols	0.004
Others	0.076
Phenols and their derivatives	1.08
Flavonoids	0.349
Non-flavonoid polyphenols	0.678
Phenolic derivatives	0.041
Others	0.007
Vitamins and their derivatives	0.47
Ascorbic acid and its derivatives	0.139
Other water-soluble vitamins	0.289
Lipid-soluble vitamins	0.030
Others	0.016
Salts	0.97
Others	5.27

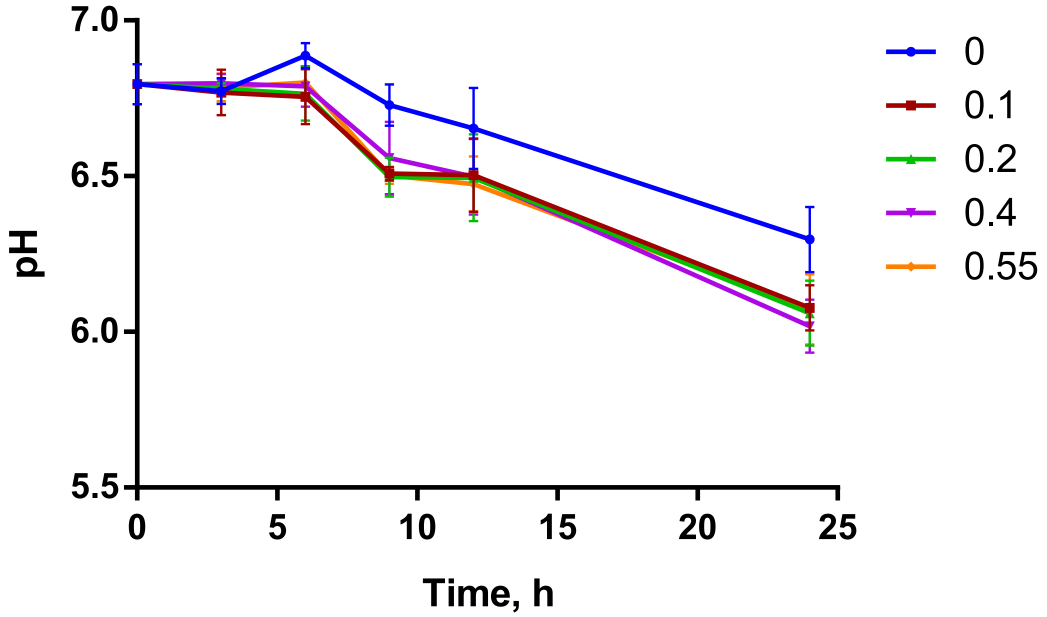

Supplementary Figure S1 Time course of the value of pH in inoculum in different treatments.

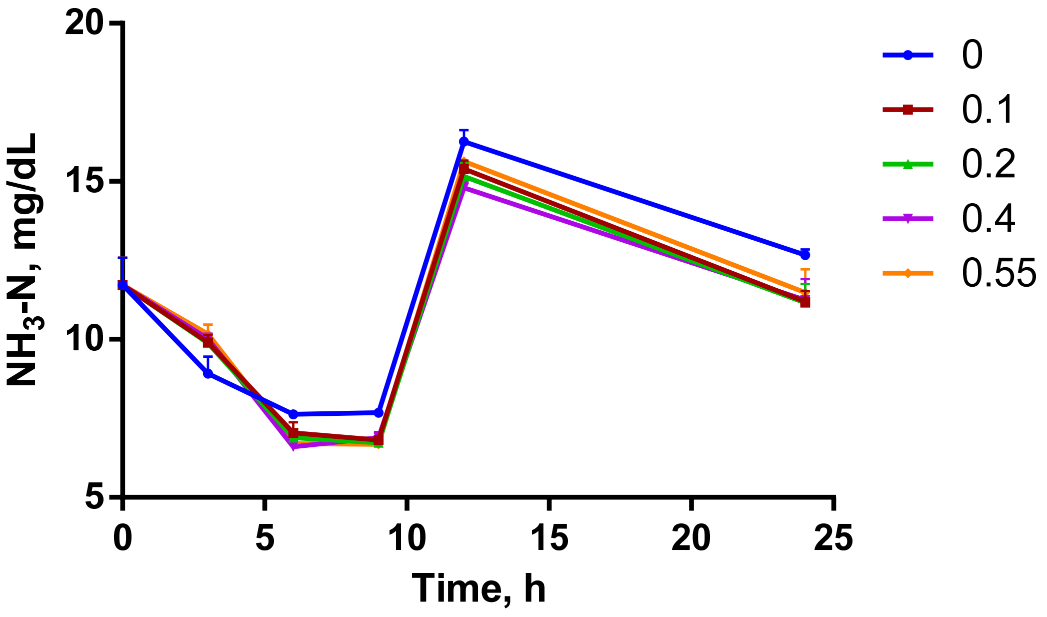

Supplementary Figure S2 Time course of the concentration of NH_3-N in inoculum in different treatments (Abbreviations: NH_3-N, ammonia nitrogen).
